# Patient, family member and healthcare professionals’ experiences of physiotherapy airway clearance techniques during hospital admission: a scoping review

**DOI:** 10.1183/23120541.00140-2026

**Published:** 2026-09-01

**Authors:** Kirsty Jerrard, Rhian Dowding, Brenda O'Neill, Judy M. Bradley, Bronwen Connolly

**Affiliations:** 1 Wellcome-Wolfson Institute for Experimental Medicine, School of Medicine, Dentistry and Biomedical Science, Queen's University Belfast, Belfast, UK; 2 School of Health Sciences, Faculty of Life and Health Sciences, Ulster University, Belfast, UK; 3 Department of Physiotherapy, The University of Melbourne, Melbourne, VIC, Australia

## Abstract

**Introduction:**

Effective secretion management is crucial for hospitalised patients with retained secretions due to critical illness, chronic respiratory disease or infection. Airway clearance techniques (ACT), primarily physiotherapist-delivered and advocated in guidelines, support secretion removal. However, limited knowledge exists regarding the experiences of patients, family members and healthcare professionals involved in ACT. The aim of the present study was to explore the experiences of patients, family members and healthcare professionals of ACT in hospital settings.

**Methods:**

Following JBI (formerly Joanna Briggs Institute) recommendations and the Preferred Reporting Items for Systematic reviews and Meta-Analyses extension for Scoping Reviews Checklist, searches were conducted in CINAHL Complete, EMBASE, MEDLINE ALL, PsycINFO and ProQuest Dissertations & Theses Global, from inception to 17 November 2025. Qualitative studies involving patients (≥16 years) receiving ACT, family members involved or healthcare professionals delivering ACT in the hospital setting were eligible. Screening and data charting were performed independently and in duplicate. Findings were synthesised descriptively and narratively.

**Results:**

Of 10 370 records identified, 11 studies met the inclusion criteria. Six studies reported primary themes related to ACT, while relevant source text from the remaining five studies was extracted and integrated into the narrative synthesis. Populations included healthcare professionals (physiotherapists, nurses, intensivists, internal medicine specialists; n=293) and patients (n=80). No studies included family members. Settings were predominantly intensive care units (ICU) (n=9), with two studies spanning ICU and ward settings. Four overarching themes emerged: three shared by patients and healthcare professionals (Knowledge, resources, and skills; Decision-making process; Perceived effects and outcomes), and one unique to healthcare professionals (Organisational factors).

**Conclusions:**

Current evidence on experiences of ACT is dominated by physiotherapists and ICU settings, with no studies capturing family perspectives and limited patient or wider multidisciplinary input across broader hospital contexts. More inclusive, stakeholder–informed research is needed to fully understand ACT use, impact and burden in clinical practice.

## Shareable abstract

**Evidence on experiences of ACT in hospital settings is dominated by physiotherapists and in the context of the intensive care unit setting, highlighting the need for comprehensive stakeholder-informed research to fully understand ACT use, impact and burden** https://bit.ly/47os9th

## Introduction

The global prevalence of respiratory diseases and infections continues to rise [1, 2] and is associated with increased hospital admissions [3] and length of stay [4]. Secretion retention is a common complication of respiratory disease and infection, and effective secretion management is therefore a crucial component of patient management, whether in the setting of the hospital ward or the intensive care unit (ICU) [5–7]. Airway clearance techniques (ACT) can be applied manually (*e.g.*, chest percussion), mechanically (using a device) or through instruction (such as breathing techniques) to facilitate secretion clearance [6], and whilst primarily delivered by physiotherapists, they may also be facilitated by other members of the multidisciplinary clinical team, *e.g.* nursing staff [8], doctors and respiratory therapists [9], as well as by family members. The physiological mechanisms underpinning ACT include airflow oscillation to mobilise secretions [6], increasing lung volumes to ventilate obstructed areas [6, 10] and increasing the velocity of expiratory airflow [6, 10]. Previous studies show that ACT are safe and feasible in chronic respiratory diseases [11–13] and critically ill populations [7, 14]. Much investigation into ACT in hospitalised patients has focused on their clinical effectiveness, determined from objective short-term outcome measures [7], often overlooking inclusion of outcomes that are also important to patients and families [15]. Few studies have examined the experience of patients, family members or healthcare professionals regarding the receipt or delivery of ACT. For patients and family members, how they perceive their role in the treatment process may be influential on treatment adherence [16] and response. Additionally, for family members, the transition to a more formal caregiving role to support healthcare interventions could result in significant burden [17]. Physiotherapists have reported adopting a patient-centred approach to delivery of ACT, with variation in clinical practice influenced by factors such as patients’ clinical presentation, physiotherapy service models, and workforce capacity and expertise [18], although these findings are limited to the ICU setting. Understanding whether the lived experiences of patients and family members align with the professional intentions of healthcare professionals is essential to achieving patient-centred care [19]. The aim of this scoping review was to map the existing literature regarding patients’, family members’ and healthcare professionals’ experiences of ACT in hospitalised adults.

**Lessons for clinicians**

- Existing studies focus primarily on physiotherapists’ experiences of airway clearance techniques and intensive care unit settings, with minimal patient input and no family perspectives.
- Four themes describing experiences of airway clearance techniques were identified that could be translated across different hospital settings.
- This review underscores the need for broader, stakeholder-informed research to fully understand the use, impact and burden of airway clearance techniques in clinical practice, which could meaningfully inform future patient care.

## Methods

The review was developed and performed according to JBI (formerly Joanna Briggs Institute) methodology and reported in line with the Preferred Reporting Items for Systematic reviews and Meta-Analyses extension for Scoping Reviews (PRISMA-ScR) Checklist [20] (supplementary material 1). A search of Open Science Framework Registries (OSF) and Figshare on 10 October 2024 identified no existing reviews on this topic. The protocol was registered *a priori* (https://doi.org/10.17605/OSF.IO/P4XJM). The research team comprised physiotherapists with methodological and clinical expertise.

### Review questions

1) What are the experiences of hospitalised adults and their family members with ACT?
2) What are healthcare professionals’ experiences with delivering ACT to hospitalised adults?

### Study eligibility

Eligibility criteria were established using the Population-Concept-Context framework (table 1). Study design included qualitative methodologies, as well as editorial, opinion papers, and narrative commentaries. These sources were included to provide contextual insights and expert perspectives that help interpret experiences of ACT in the hospital setting. Quantitative study designs were excluded. Mixed methods studies were eligible if they contained qualitative data. Studies published in any language were eligible.

**TABLE 1:** Scoping review eligibility criteria

Component	Inclusion	Exclusion
**Population**	Adults aged ≥16 years in hospital receiving ACT delivered by a healthcare professional Family members, defined as any individual who provides support or care to a patient in hospital, who observed or assisted in the delivery of ACT Healthcare professionals, of any background, involved in the delivery of ACT	
**Concept**	Experience or perspective of delivering or receiving ACT by a trained individual ACT including, but not limited to, positioning, hyperinflation techniques, manual techniques and mechanical insufflation–exsufflation. Saline instillation and suction procedures were considered if used as an adjunct to an ACT	Respiratory muscle training interventions Usual airway clearance management practices in isolation, such as suctioning or humidification Self-directed ACT performed independently by patients without input from a trained individual (*e.g.* unsupervised deep breathing exercises)
**Context**	Hospital setting	Non-hospital settings

ACT: airway clearance techniques.

### Search strategy and information sources

A search strategy was developed, in collaboration with a specialist health science librarian, to identify published and unpublished/grey literature (supplementary material 2). Five databases were searched from inception to 27 November 2024: CINAHL Complete, EMBASE, MEDLINE ALL, PsycINFO and ProQuest Dissertations & Theses Global databases. Manual searches of reference lists of included studies and the study team's personal libraries (using keywords from the main search strategy) were also conducted to identify additional potentially eligible studies.

### Study selection

Retrieved citations were initially imported into EndNote 21 (Clarivate Analytics, PA, USA) to remove duplicate and irrelevant records based on their title by one reviewer (K. Jerrard). Remaining results were imported into Covidence (Veritas Health Innovation, Melbourne, Australia) for screening. Title and abstract screening were piloted independently by two reviewers (K. Jerrard and R. Dowding) using a random sample of 25 records. Following the pilot, all remaining titles and abstracts, and full texts were then screened independently by the same reviewer pair (K. Jerrard and R. Dowding). Any disagreements were resolved through discussion, with third-party arbitration when needed (B. Connolly).

### Data charting process and analysis

Data were extracted using a predefined data charting template (supplementary material 3), piloted and completed independently by two reviewers (K. Jerrard and R. Dowding). The same reviewer pair verified the data by cross-checking the charting tables. Data were summarised using descriptive statistics (frequencies and proportions) and narrative synthesis [20, 21]. The latter process involved familiarisation with each study to extract primary themes relating to ACT, followed by a comparative process highlighting similarities and differences across studies. Primary themes from each study were then grouped conceptually and overarching themes with accompanying descriptors developed, and agreed by the study team, to reflect these groups. Verbatim illustrative quotes from the studies were used to provide contextual depth. All studies reporting primary themes were included in the synthesis to ensure inclusive representation of the evidence base. Studies that did not report primary themes did not contribute to the development of the overarching descriptors; however, relevant text data were extracted and assigned to the most appropriate overarching theme where they contributed to the refinement of theme descriptors.

For mixed-methods studies, data extraction solely focused on the qualitative components. Studies conducted across hospital and non–hospital settings were included only when hospital–specific data could be clearly identified; mixed patient populations within hospital settings were eligible. No assessment of methodological rigour was conducted in keeping with scoping review methodology [20].

## Results

### Study identification

**FIGURE 1:**
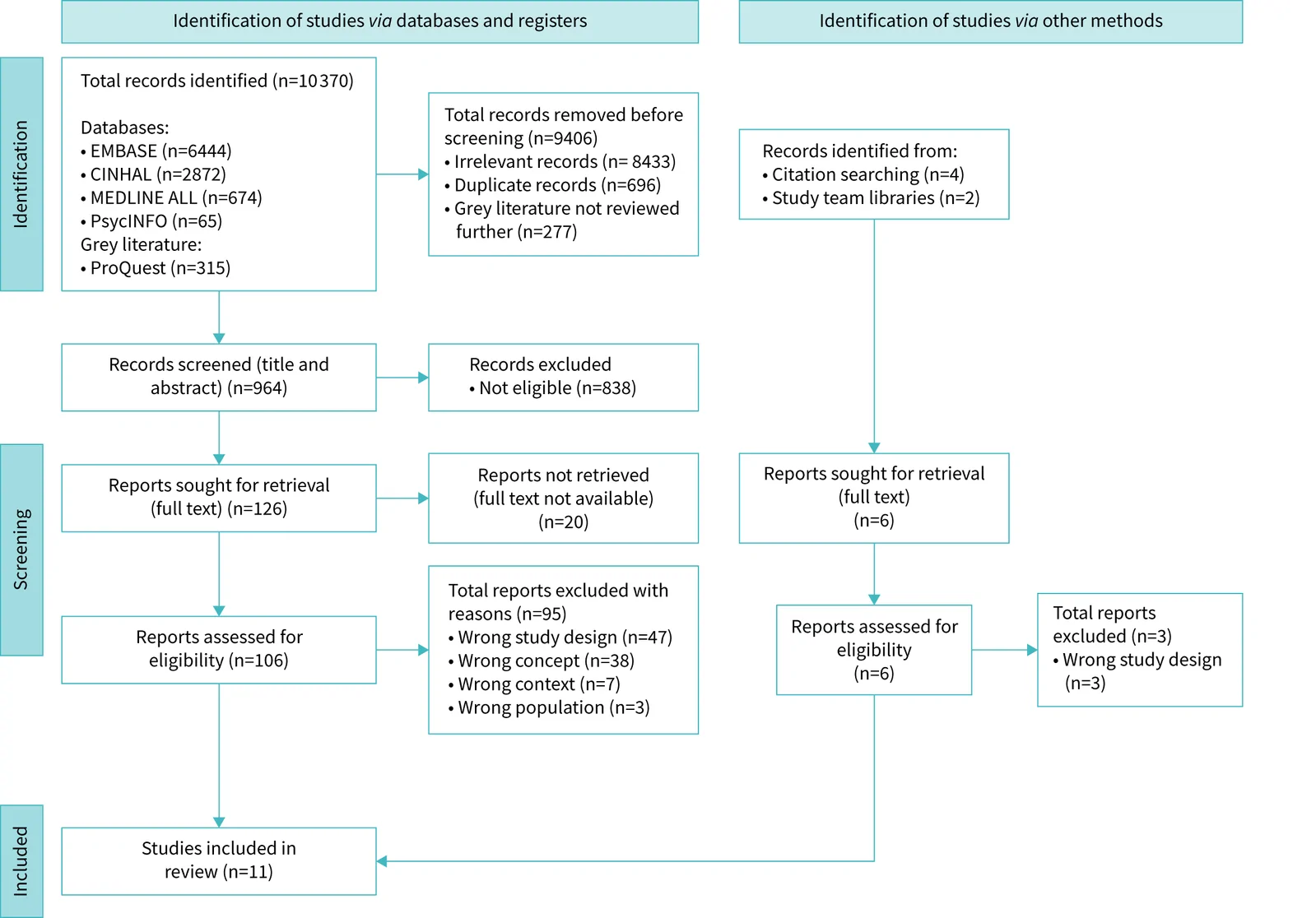
PRISMA flowchart of study selection process. Flow diagram showing the identification, screening, eligibility and inclusion of studies in the review, based on PRISMA 2020 guidelines.

Searches identified 10 370 records, of which 11 studies met the eligibility criteria (figure 1). The main exclusion reasons were wrong study design (n=47) and wrong concept (n=38), based on predefined inclusion criteria (supplementary material 4).

### Characteristics of included studies

Characteristics of included studies are detailed in table 2. Of the 11 included studies, published between 2017 and 2025, seven adopted qualitative [18, 22–27], three employed mixed methods [28–30] and one was an opinion piece [31]. Studies were conducted across eight countries, most frequently Australia (n=4) [24, 27, 29, 30], and ICU was the predominant setting across all studies, with two studies also including patients from ward settings [22, 31]. Two studies related to patient experiences [23, 31], and nine studies focused on healthcare professionals’ experiences or perspectives, predominantly physiotherapists [18, 22, 24, 28–30]. No included studies investigated family members’ experiences. Across the 11 included studies, four examined physiotherapy practice broadly which included the respiratory management of patients [23, 26, 28, 31], six reported on a variety of ACT, *e.g.*, positioning, manual techniques and hyperinflation techniques [18, 22, 24, 27, 29, 30], and one focused specifically on the use of a mechanical insufflation-exsufflation (MI-E) device [25]. Healthcare professionals included physiotherapists, nurses and medical staff with experience in acute and critical care settings. Physiotherapists worked across general, cardiac and neurological ICUs, as well as acute hospital wards, and represented clinical, academic and specialist roles. Reported physiotherapy experience ranged from 3 to 27 years. Nursing participants included ICU nurses, clinical nurse specialists and nurses with additional ventilation–specific training, typically with at least 2 years’ clinical experience. Medical staff included internal medicine specialists and consultant intensivists with experience working in ICU. Patients included individuals admitted to a surgical ICU following emergency surgery or trauma and patients hospitalised with COVID–19 in the ICU or acute care units.

**TABLE 2:** Summary of the characteristics of included studies

First author, Year Country	Aim(s)	Methodology Data collection Setting	Summary of main findings	Population Characteristics Sample size
**Cakmak, 2019 [28]** **Turkey**	To 1) identify the characteristics of physiotherapy practice and 2) determine barriers towards applying physiotherapy in ICUs in Turkey	Mixed methods Survey ICU	Common reasons for physiotherapy referral in ICU included atelectasis, pneumonia/lung infection, acute respiratory failure, post-operative cardiovascular surgery and COPD exacerbation. Frequently used techniques were positioning, active exercises, breathing exercises, passive exercises and percussion Barriers to physiotherapy were related to physiotherapist factors, patient factors, teamwork, equipment and legal issues	Healthcare professionals Physiotherapists working in the ICU who are part of the Turkish Physiotherapy Association network (n=65)
**Connolly, 2020 [18]** **UK**	To explore and describe current UK physiotherapy practice relating to ACT and mucoactive agents in critically ill adult patients with acute respiratory failure in the ICU	Qualitative Semi-structured focus groups (led by an experienced qualitative researcher) ICU	Five themes related to ACT were identified: repertoire of ACT, staffing and skillset, commencing respiratory physiotherapy, technique selection, and determining effectiveness. A summary of key features of standard practice was developed	Healthcare professionals Physiotherapists with 3–27 years of critical care experience; 10 worked in general (mixed medical, surgical, trauma, severe respiratory failure services) ICUs, four in cardiac ICUs and one in a neurological ICU; ICU bed capacity ranged from 16 to 54 beds (n=15)
**Eggmann, 2021 [22]** **Switzerland**	To describe the experience of Swiss physical therapists in the treatment of patients with COVID-19 during their acute care hospital stay and to discuss challenges and potential strategies in the clinical management of these patients	Qualitative Case report series ICU and acute wards across five Swiss hospitals	Physical therapists treated COVID-19 patients in ward and ICU settings using patient education, prone positioning, early mobilisation and respiratory therapy. Patients were often unstable with slow recovery requiring individualised strategies. Common complications included severe weakness, post extubation dysphagia, weaning failure, or presenting with anxiety or delirium	Healthcare professionals Physiotherapists treating patients with COVID-19 during their acute care hospital stay (n=not reported)
**Karachi, 2023 [23]** **South Africa**	To describe patient perceptions and satisfaction regarding the physiotherapy services and care received during their stay in a surgical ICU	Qualitative Semi-structured interviews (led by the principal investigator, an independent physiotherapist not affiliated to the physiotherapy department or involved with patient treatment) 14-bed surgical ICU at a tertiary institution in the Western Cape province of South Africa	Three themes were identified: linking therapy to clinical outcome, the importance of developing a trusting relationship and communication	Patients Admitted for emergency surgery and trauma, with a median age of 44 years and median ICU length of stay of 6 days (n=18)
**Raios, 2024 [24]** **Australia**	To determine physiotherapists’ current practices and perspectives regarding their role in caring for people who are potential lung donors in the ICU	Qualitative Semi-structured focus groups (guided by an experienced facilitator) ICU	Six key themes were identified: 1) physiotherapists’ involvement in care was highly variable; 2) physiotherapists were not aware of existing evidence or guidelines for the care of people who are potential donors and followed usual practices; 3) a consistent vision of the physiotherapy role was lacking; 4) physiotherapists’ engagement with the team routinely involved in care of people who are potential donors varied considerably; 5) physiotherapists faced practice challenges associated with delivering care to potential donors; and 6) several enablers could support a role for physiotherapy in this patient population	Healthcare professionals Physiotherapists with a mean age of 33.5 years, 89% were female with a median 7.5 years of ICU experience at six metropolitan health services in Victoria, Australia (n=27)
**Stilma, 2023 [25]** **the Netherlands**	To understand perceived barriers and facilitators as well as detailed data on when and how MI-E is initiated, escalated, de-escalated and discontinued	Qualitative Semi-structured focus groups (led by a moderator and assistant moderator who undertook additional training in qualitative research) ICU	Experience with MI-E varied from infrequent to frequent across different patient populations. Four key themes emerged: knowledge, beliefs, clinical decision-making and future adoption	Healthcare professionals with experience of MI-E (n=35)^#^ · ICU nurse (n=7) · ICU nurse with additional 14-month ventilation course (n=9) · Physicians (intensivists) (n=3) · Physiotherapists (n=6) · Clinical nurse specialist (n=2)
**van der Lee, 2017 [29]** **Australia**	To identify the degree of variability in physiotherapy practice for intubated adult patients with CAP and to explore ICU physiotherapist perceptions of current practice for this cohort and factors that influence physiotherapy treatment mode, duration and frequency	Mixed methods Online survey ICU	The key rationales for providing a respiratory intervention were improved airway clearance, alveolar recruitment and gas exchange. Clinical practice was varied but common techniques included positioning and use of hyperinflation techniques. Treatment duration was influenced by sputum volume, viscosity and purulence, cough effectiveness, chest X-ray and auscultation. Workload and staffing affected intervention duration and frequency	Healthcare professionals Physiotherapists across 88 Australian public and private hospitals, with a minimum of 5 years of clinical experience and a minimum 12 months of experience as a senior physiotherapist working in an adult ICU within the last 5 years (n=75)
**van der Lee, 2018 [30]** **Australia**	To determine expert consensus for best physiotherapy practice for invasively ventilated adults with CAP	Mixed methods Modified online Delphi survey consisting of three rounds ICU	Outcome achieved was 38 consensus statements covering assessment and treatment	Healthcare professionals Physiotherapists with a minimum of 5 years as a qualified physiotherapist and a minimum of 3 years of experience in critical care. Participants represented clinical, academic and specialist roles (n=29)
**van der Lee, 2020 [27]** **Australia**	To conduct multidisciplinary peer review of expert consensus statements for respiratory physiotherapy for invasively ventilated adults with community-acquired pneumonia, to determine clinical acceptability for development into a clinical practice guideline	Qualitative Focus groups (conducted by the primary investigator (a trained moderator) and a note-taker) ICU	Interprofessional teamwork and communication, patient safety and culture were key contextual factors influencing the application of the statements in physiotherapy practice and across different ICU settings	Healthcare professionals with more than 5 years’ experience and working in an Australian level 2 or 3 ICU (n=26) · Physiotherapists (n=16) · Nurses (n=4) · Consultant intensivists (n=6)
**Bellone, 2025 [31]** **Italy**	To retrospectively describe the functional outcomes associated with an early, structured, multidisciplinary rehabilitation programme administered to hospitalised patients with acute SARS-CoV-2 infection. A secondary aim is to explore potential associations between rehabilitation variables (*i.e.*, timing, number of sessions, early approach) and improvements in functional status	Opinion paper From retrospective data ICU and acute care units	Early structured multidisciplinary rehabilitation during acute hospitalisation for COVID-19 patients improved functional recovery. The approach shows potential applicability to chronic respiratory disease management	Patients COVID-19 patients hospitalised in the ICU (n=25) and acute care units (internal medicine, pulmonology, infectious diseases departments, n=37) between March and June 2020
**Solares-Mogollón, 2025 [26]** **Spain**	To describe the strengths and barriers of administering physiotherapy treatment to patients admitted to an ICU as perceived by healthcare professionals with experience in these units	Qualitative Interviews ICU	Healthcare professionals emphasised the importance of early physiotherapy, noted benefits for respiratory and functional recovery. Identified barriers included insufficient staffing, high patient-to-physiotherapist ratios and communication challenges within the multidisciplinary team	Healthcare professionals from an ICU, with at least 2 years of clinical experience and who currently work or have worked in coordination with physiotherapists within the ICU · Physiotherapist (n=7) · Nursing (n=7) · Internal medicine specialist (n=7)

Aims are reported verbatim as per the source. ICU: intensive care unit; COVID-19: Coronavirus disease 2019; ACT: airway clearance techniques; MI-E: mechanical insufflation–exsufflation; CAP: community-acquired pneumonia. ^#^: for Stilma *et al.* (2023), professional subcategories are reported as described in the original source and do not sum to the total number of healthcare professionals with experience of MI‑E (n=35).

### Narrative synthesis

**FIGURE 2:**
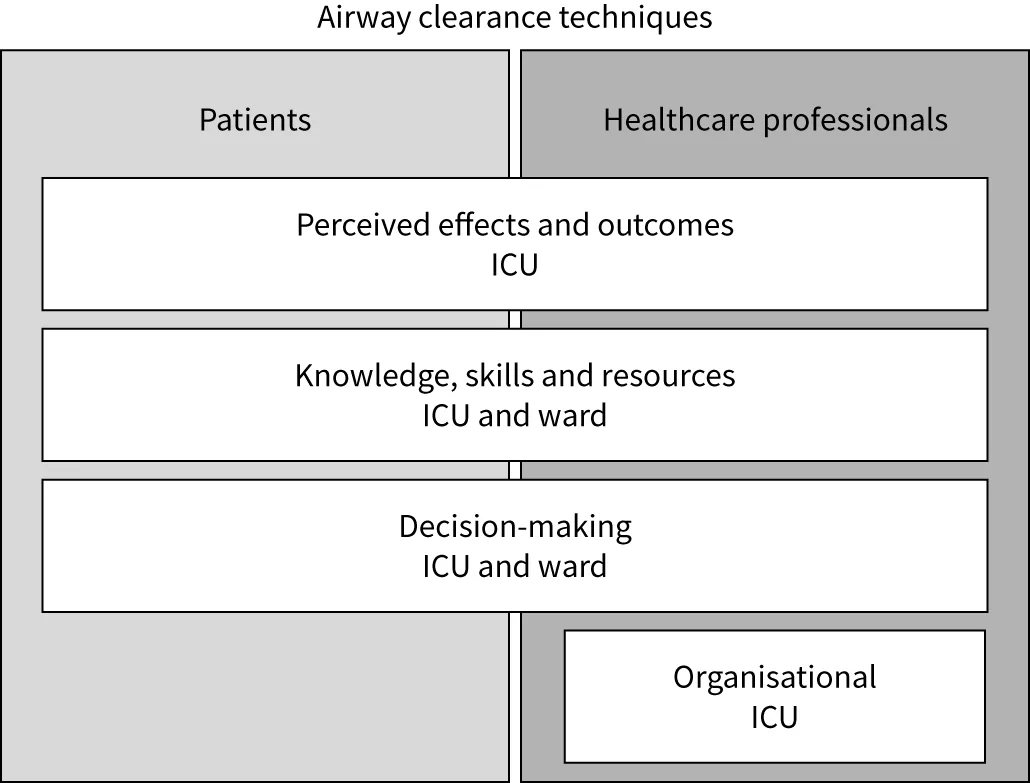
Summary of experiences of airway clearance techniques from the perspectives of patients and healthcare professionals. The figure depicts the identified themes from studies on airway clearance techniques from the perspectives of patients and healthcare professionals. It highlights both commonalities and differences in their experiences. The text beneath each theme identifies the clinical settings in which it was found.

Data extraction of primary themes relevant to the review question was conducted on six of the included studies [18, 24, 25, 27, 29, 30], which were analysed as part of the narrative synthesis. Five studies [22, 23, 26, 28, 31] did not explicitly report primary themes related to ACT, therefore relevant data were extracted and overarching themes were assigned to ensure consistency and integration into the narrative synthesis (table 3). Data extraction is available in supplementary material 5. Four common themes related to experiences of ACT were identified (figure 2). Three themes were common across both patients and healthcare professionals: knowledge, resources and skills, decision-making process, and perceived effects and outcomes. Only two included studies related to patient experiences resulting in relatively limited patient–derived data underpinning these shared themes. The fourth theme was unique to healthcare professionals: organisational factors.

**TABLE 3:** Summary table describing the themes identified through narrative synthesis of findings from included studies

Themes, descriptions and related studies	Example text
**Themes common to patients and healthcare professionals**	
Theme: Knowledge, resources and skills [22–25, 28, 30]	“Manual compressions on the chest and abdomen were performed with just enough intensity to modify expiratory flow to aid secretion clearance.” [22] “Physiotherapists’ professionalism, reassurance and communication made patients feel comfortable and safe, thus building a trusting relationship with the physiotherapists.” [23] “Physiotherapists were unaware of empirical evidence or guidelines specific to respiratory physiotherapy interventions for potential donors.” [24]
Description: The required understanding, training and practical competency required for ACT use, including an understanding of the current evidence base and gaps within it, and awareness of relevant clinical governance (where applicable), awareness of guidelines, access to educational resources, and technical and interpersonal skills	
Theme: Decision-making process [18, 22, 25, 27, 29–31]	“Participants described a range of ACT, for use in isolation or combination, and adapted these according to clinical presentation.” [18] “Participants indicated that haemodynamic instability is common early in the ICU admission for this cohort. A conversation with the bedside nurse would be an integral part of assessment of haemodynamic stability.” [27] “Respiratory rehabilitation focused on improving deep lung ventilation and secretion clearance.” [31]
Description: How healthcare professionals choose to use and modify ACT by gathering patient-related information from different sources, performing individualised assessments, implementing ACT and modifying them according to patient status, whilst mitigating potential risks of harm in the absence of strong evidence for their use	
Theme: Perceived effects and outcomes [18, 23, 26]	“Outcome measures for evaluating effectiveness of ACT included subjective…and objective.” [18] “Patients described various activities during ICU physiotherapy, including chest physiotherapy…which some felt helped with breathing and rib pain.” [23] “Better management of secretions.” [26]
Description: Patient-reported benefits of ACT as well as healthcare professional-reported subjective and objective outcome measures used to assess their effectiveness	
**Themes unique to healthcare professionals**	
Theme: Organisational factors [18, 24–28]	“Physiotherapists had differing opinions about their role in the care of potential donors.” [24] “Physiotherapists in the Sydney group were more inclined to treat patients in the horizontal side-lying or HDT positions and were more likely to use chest wall vibrations or percussion to assist with secretion clearance.” [27] “Practically all health professionals were in agreement about the knowledge of the physiotherapist's functions regarding the development of respiratory techniques and motor work in patients.” [26]
Description: The impact of workplace culture and environment on the delivery of ACT, such as the use of specific ACT across different hospitals within the same country, healthcare professional expectations of staff roles and skills, physiotherapists’ involvement and collaboration with multidisciplinary teams, and limited clinical exposure to certain patient populations	

ACT: airway clearance techniques; ICU: intensive care unit; HDT: head down tilt.

#### Knowledge, resources and skills

Six studies reported on the knowledge, resources and skills required for ACT use. This included an understanding of the current evidence base and gaps within it, awareness of guidelines and relevant clinical governance, access to educational resources, and the technical and interpersonal skills needed for ACT delivery.

Knowledge underpinning ACT use was identified in several studies. One ACT described was the use of an MI-E device [25]. Physiotherapists acknowledged MI-E settings could be modified, for example to improve flow bias and mobilise secretions [25]. Physiotherapists also highlighted the limited evidence supporting the use of MI-E in mechanically ventilated patients, meaning practice was primarily guided by protocols and information from home ventilation centres (where greater evidence was available) and the lack of evidence for other ACT in the ICU setting [24, 25]. This was reflected in one physiotherapist's statement: “…I'm not aware of any…primary evidence which supports the role of respiratory physiotherapy in ICU patients for donation.” (Healthcare professional – Physiotherapist) [24] Physiotherapists working with potential lung donors faced additional challenges, such as uncertainty about the legal and ethical frameworks guiding clinical practice [24].

With regard to resources, access to ACT-related education and support was a concern raised. In the ICU setting, healthcare professionals (nurses, physiotherapists and doctors) reported MI-E use was supported by training nursing staff to be experts in its application and the provision of annual education sessions. They also highlighted inconsistent training and safety information from MI-E companies, noting this could create challenges when patients using MI-E transitioned between settings, due to a lack of support [25]. ICU physiotherapists emphasised the need for increased ICU-specific training during undergraduate education, as well as access to experienced physiotherapists for practical training in the clinical environment [28]. One physiotherapist commented: “…working in the ICU without the presence of an experienced physiotherapist initially made me insecure about my practical experience…supporting education through undergraduate and post–graduate studies may strengthen physiotherapy practice.” (Healthcare professional – Physiotherapist) [28].

Technical skills for ACT delivery were also discussed. Despite awareness of how MI-E could be adjusted, physiotherapists in the ICU relied on pre-programmed settings due to limited device exposure and a lack of skills in its use. Outwith application of MI-E, physiotherapists emphasised the importance of their specialist skills in using a variety of ACT (breathing exercises, active cycle of breathing techniques, positioning, mobilisation, manual chest wall techniques, inspiratory positive pressure breathing, autogenic drainage, high-frequency chest wall oscillation, recruitment manoeuvres and saline instillation) to mobilise distal airway secretions [22, 24, 30]. These specialist skills were distinct from suctioning used in isolation, employed generically to clear secretions primarily from the upper airways. This distinction was illustrated by one physiotherapist who noted: “Suctioning only clears the proximal airways…there are physiotherapy techniques which can assist with sputum transport from the distal to proximal airways, and may make suctioning more effective, and also techniques which can limit or reverse some of the detrimental effects of suctioning.” (Healthcare professional – Physiotherapist) [30].

Additionally, interpersonal skills employed by healthcare professionals were viewed as central to supporting patients receiving ACT. Patients in the surgical ICU described how physiotherapists’ professionalism and communication style was key to developing a trusting relationship and shaping their interactions [23]. Those who had positive experiences with their physiotherapists reported a feeling of comfort and safety. In the ICU setting, physiotherapists reported communication with patients could be challenging due to factors such as patient instability, pain or delirium, which affected their ability to cooperate [28]. One patient described how the presence of “pipes” made communication challenging, and highlighted the physiotherapist's efforts to support communication: “…it was difficult…there was no other way for me to communicate with her…she brought the book and pen every day so I could write…she always asked if I had questions…the message got across, and I could understand….” (Patient) [23].

#### Decision-making process

Healthcare professionals described variability in how decisions to commence ACT were made. In the ICU these decisions were grounded in individualised clinically reasoned assessments [18] to determine which patients would benefit from, or required escalation of ACT [30]. This approach was reflected in one physiotherapist's account: “If there's no indication from airway clearance…ventilation is by minimal oxygen, no real secretion issues, then we won't actually get involved until somebody is identified…." (Healthcare professional – Physiotherapist) [18]. Evaluating information from multiple sources, including discussions with bedside nurses and medical staff regarding clinical stability of patients, was common in the ICU [27]. Other important considerations influencing ACT use included subjective clinical judgement of the effectiveness of other clearance methods, cough strength, swallow function, fatigue levels and secretion tenacity; the latter being particularly emphasised by ICU physiotherapists [18, 22, 30].

Physiotherapists reported having a range of ACT available in the ICU setting which could be used individually or in combination and adapted as needed [18], with interventions centred on optimising secretion clearance, alongside improving lung compliance and ventilation [29]. For example, some senior physiotherapists preferred manual hyperinflation particularly when treating patients who were conscious, agitated or found ventilator hyperinflation difficult to tolerate [29]. As one physiotherapist explained: “…it can allow more precise co–ordination with the patient's breathing pattern in patients who are awake and agitated or intolerant of ventilator hyperinflation…it provides better feedback regarding lung compliance and secretion load.” (Healthcare professional – Physiotherapist) [29] In contrast, other senior physiotherapists favoured ventilator hyperinflation for its ability to maintain positive end-expiratory pressure and provide accurate monitoring of respiratory parameters, perceiving it as comparable to manual hyperinflation in its effects on lung compliance, oxygenation and airway clearance [29]. Patients with COVID-19 in the ICU experienced respiratory interventions designed to support deep breathing and facilitate secretion clearance. In the ward setting, their experience shifted towards strategies centred on lung recruitment, supported by use of volume-based ACT utilising deep, slow inspirations [31].

Patient safety was central to ACT-related clinical decision-making in both ICU and ward settings. In the former setting, examples included stopping enteral feeds before placing a patient in a head-down tilt position [30], adjusting MI-E settings in ventilated patients to mitigate potential harm [25], and using ICU displays to monitor and adapt ACT according to the patient's response [22]. This emphasis on safety was echoed by one physiotherapist who reported: “…I would only perform head–down tilt during physiotherapy treatment…and only if there were no other indications that would put the patient at risk…feeds would be stopped during this time as well.” (Healthcare professional – Physiotherapist) [30] On the ward, physiotherapists applied positioning techniques slowly and adapted breathing techniques to avoid the patient experiencing respiratory deterioration [22].

#### Perceived effects and outcomes

ACT effectiveness and outcomes were reported from both patient and healthcare professional perspectives. Awake and alert ICU patients, which included those admitted for elective, emergency or trauma-related reasons, described benefits such as improved breathing, reduced rib pain and relief following chest physiotherapy, breathing exercises or use of a positive expiratory pressure (PEP) bottle [23]. One patient commented: “…they learn me how to blow that bottle…there's no pain any more in my ribs.” (Patient) [23].

From the perspective of healthcare professionals, enhanced section management in patients receiving respiratory physiotherapy was reported [26]. This was reflected in one nurse's description of physiotherapy input as “…Better management of secretions” (Healthcare professional – Nurse) [26]. Physiotherapists described using a combination of subjective and objective measures to assess ACT effectiveness [18]. Subjective measures included visible secretions cleared and effectiveness of the patient's cough, while objective measures involved monitoring short-term physiological responses, such as oxygen levels, and reviewing chest radiographs for radiological changes.

#### Organisational factors

Organisational factors, related to workplace culture and the ICU environment were reported exclusively by healthcare professionals in the ICU and were described as influencing ACT use. Physiotherapists reported variation in physiotherapy service models could lead to differences in staffing levels and skill mix, influencing clinical practice [18]. Non-ICU-specialist and/or less experienced staff, particularly when working on-call or during weekends, were perceived to use a more restricted range of ACT than specialist ICU or more experienced physiotherapists [18]. One physiotherapist explained: “Our on–call rotas are for all of our physio team barring the outpatient physios, so we really struggle to get that more technical treatments going over the weekend.” (Healthcare professional – Physiotherapist) [18] A lack of full-time, dedicated physiotherapy staffing within the ICU was also a reported challenge [28], as well as inter-ICU variability in approach to ACT use, *e.g.* healthcare professionals in one ICU empirically adopting use of one manual ACT compared to healthcare professionals in another, despite being in the same national healthcare jurisdiction [27].

Expectations of staff roles and the extent of multidisciplinary collaboration also shaped organisational practice. ICU consultants and nurses described the importance of effective collaboration with physiotherapists and other multidisciplinary team (MDT) members to ensure coordinated high-quality patient care [27]. This was demonstrated in one nurse's account: “it needs to be a decision that's made in conjunction with medical and nursing teams …it should be a team decision, not a unilateral decision…” (Healthcare professional – Nurse) [27]. In its absence, *e.g.* where physiotherapists demonstrated uncertainty regarding their role with particular specialist patient groups, this led to a lack of advocacy for the physiotherapy role and inconsistent representation within the ICU MDT [24]. Other ICU physiotherapists highlighted that they often faced challenges due to a lack of awareness and understanding of their role within the MDT, which affected initiation or timing of physiotherapy treatments [28]. In contrast, a study involving physiotherapists, internal medicine specialists and nurses found that nearly all health professionals recognised the physiotherapist's expertise in using ACT to manage secretions [26].

Organisational influences were also reflected in how teams adopted specific ACT. One study investigating the importance of MDT collaboration (involving doctors, nurses and physiotherapists with ICU experience), described how the team's collective knowledge, review of the evidence base and shared training influenced the adoption of specific ACT such as MI-E [25]. This was illustrated by one participant who explained: “We had to look at the research and if there was not good research, thus our professors say: No we do not do it for intensive care patients.” (Healthcare professional) [25].

## Discussion

This review aimed to explore the existing literature on patients’, family members’ and healthcare professionals’ experiences of ACT in hospitalised adults. No included studies reported family member experiences, and findings reflect only patient and healthcare professional perspectives. Four themes influencing ACT were identified across the included studies, with three being consistently reported by both patients and healthcare professionals, albeit their relevance varying across groups. Most studies in this review were published in the last 5 years [18, 22–27, 31], focused exclusively on physiotherapists’ views [18, 22, 24, 28–30] and the ICU setting [18, 23–30]. Potential geographical bias, with the majority of studies being conducted in Australia, should be considered when interpretating the findings. Contextual factors, such as healthcare infrastructure and clinical guidelines, may influence the applicability and transferability of results to other healthcare jurisdictions. Overall, our findings reflect a growing recognition of the importance of understanding the perspectives and experiences of those involved in ACT. However, further research which is representative of all stakeholders’ experiences of ACT across a broader range of hospital settings is required.

The first theme, knowledge, resources and skills, was shared across patients and healthcare professionals. Clinical guidelines within hospital settings can improve patient outcomes and consistency of care [32–34]. For example, European guidelines for ACT in patients with bronchiectasis, identify ACT as a core component of patient management [35], while ICU guidelines recommend a personalised approach to ACT delivery in patients [36]. Nonetheless, evidence is scarce addressing ACT implementation in hospitalised patients outside of the ICU setting, particularly those experiencing acute exacerbations of chronic respiratory disease. Furthermore, even where guidelines exist (such as for ICU), limited underpinning evidence precludes translation into specific clinical practice protocols [36]. The continued use of ACT practice in its current form within this setting highlights its empirical nature [7, 37, 38]. This experience-based, rather than evidence-based, model of practice may also extend to other acute hospital environments. Clinical practice is also governed by legislative and ethical policies [39–41]. Knowledge of these, however, and their influence on professional responsibilities, may be limited [24], albeit this scenario is not unique to the context of ACT and is a wider issue among UK healthcare professionals [42, 43].

ICU standards in the UK and Australia and New Zealand recommend that physiotherapy is available 24 h a day, 7 days a week [36, 44], but implementation varies. In the UK, hospitalised patients generally have access to round-the-clock respiratory physiotherapy, while only about half of ICUs in Australia and New Zealand offer on-call physiotherapy services [45]. European surveys also highlight limited overnight physiotherapy availability in the ICU setting [46]. Regardless of availability patterns, respiratory physiotherapy in these contexts is often delivered by a mix of respiratory specialist and nonspecialist physiotherapists [36, 45, 47]. To address this, efforts have been made to support physiotherapists to help identify training needs [48], and establish service recommendations for acute respiratory and on-call care [47]. National and international initiatives have established the minimum competencies for physiotherapists working in ICU of which a relatively small proportion (12–16%) specifically address ACT reflecting the highly specialised nature of these interventions [49, 50]. In the UK, this is supported by a nationally recognised capability framework [51].

Future work exploring implementation and adaption of these frameworks, including in other hospital contexts, healthcare jurisdictions and clinical specialities (*e.g.*, chronic respiratory disease), will be important in maximising their impact and ability to enhance clinical practice. Standardisation of competencies could also support consistent technical training and associated resources which our study highlighted are lacking. Simulation-based education offers a promising approach at pre-registration and postgraduate levels by giving physiotherapists the opportunity to develop and maintain technical respiratory skills in a safe environment through experiential, real-life scenarios [52].

Interpersonal skills were also an important component of the knowledge, resources and skills theme. Standards of healthcare professional behaviour are essential for fostering trust and confidence among patients, colleagues and the public [53–56]. They also guide the maintenance of professional boundaries, effective communication and patient engagement in treatment. However, some patients in the acute setting face communication barriers and reduced autonomy due to illness severity [28, 57] requiring proactive facilitation to support involvement in treatment choice [58]. Clear communication with family members would be important in these situations, offering insights into a patient's wishes and guiding care when patients are unable to participate [59]. Poor communication can lead to patients and family members feeling disconnected and excluded from shared decision-making [60]. This reinforces the value of healthcare professionals dedicating time to foster meaningful relationships that respond to the individual needs of patients and family members. That our review did not identify any studies including family members’ perspectives underscores the need for this to be addressed in a more purposeful and focused approach in the future.

The second theme, decision-making process, reflected the complex nature of ACT delivery. For healthcare professionals delivering ACT, decision-making is complex and dynamic, influenced by multiple sources of information and evidence. In one conceptual model related to physical therapy, healthcare professionals were described as needing to make decisions quickly and efficiently, especially in situations where time is limited or evidence is absent [61]. This is applicable to ACT delivery which is often time-critical in the acute environment. However, less experienced physiotherapists may lack sufficient experience or intuition to make decisions as quickly or effectively as their more experienced counterparts [61]. Training combining core technical and interpersonal skills, knowledge and situational awareness, while considering the contextual factors of the hospital setting in which they are applied, could support complex care decisions in this patient cohort and help develop clinical judgement skills [62]. In our review, ensuring patient safety was also a key consideration for healthcare professionals when delivering ACT, potentially reflecting an unintentional response to the varying levels of clinical experience and associated risks. In clinical practice, ensuring all healthcare professionals (regardless of level of experience) are equipped to manage the complexity and urgency of ACT-related decision-making is essential to safeguarding both patients and healthcare professionals.

The third theme, perceived effects and outcomes, highlighted variation in how effectiveness was judged across stakeholders. Disparity between patients’ and healthcare professionals’ judgements of ACT effectiveness highlights the importance for outcomes that are appropriate and meaningful for all relevant groups. Core outcome sets (COS) represent one methodological approach to achieving consensus on priority outcomes across different stakeholders (*e.g.* patients, family members, healthcare professionals, researchers) [63] and can be used in research and clinical contexts. For airway clearance strategies, COS have recently been developed specifically for trials involving mechanically ventilated critically ill adults [64], community patients with neuromuscular disease [65] and individuals with bronchiectasis [66]. Whilst valuable, these COS primarily focus on stable or community phases of care. During severe exacerbation, ACT may be used intensively, and future COS should consider the needs of patients across all phases of care and disease trajectory. It is also important to note that, although these COS have been designed to incorporate patient and family member perspectives, the extent of involvement varies. In one COS patients and healthcare professionals contributed in equal numbers during the identification phase and consensus phase, demonstrating strong stakeholder engagement [66]. However, in another COS, patient representation during consensus–building rounds was limited from the start and declined during latter stages, and family member participation was minimal [65]. As a result, patient and family perspectives were not always represented to the same extent as those of healthcare professionals. Participation may be further restricted by the frequent exclusion of non–English–speaking patients and families due to limited availability of translation services [65, 66]. Together, these factors suggest that, despite efforts to include all stakeholders, existing COS may not fully capture the priorities and lived experiences of both patients and family members. There may also be potential to translate elements of these COS to broader acute hospital populations/settings. For example, outcomes related to physiological measures such as cough strength or patient-reported effectiveness of secretion clearance could be relevant to various patients in the hospital setting receiving ACT. However, our findings suggest patients’ perspectives regarding the perceived benefits of ACT are individualised and may not be condition-specific. This highlights the importance for healthcare professionals to continually review and adapt ACT delivery to align with a patient's recovery trajectory, symptom burden and overall well-being.

The final theme, organisational factors, was reported solely by healthcare professionals in the ICU setting, primarily physiotherapists. This limits our understanding of how such factors might affect ICU patients and family members, as well as patients, family members and healthcare professionals in other hospital contexts. Whilst there may be some translational applicability of our current findings, this clearly represents an area for future investigation. In the ICU, determining an optimum staffing infrastructure (*e.g.* number, seniority, experience level, operational days) to reflect the complexity of the patient population is challenging and remains undefined [67]. Significant work has described existing services, roles and responsibilities of physiotherapists (along with therapy staff from other disciplines) [68–70], albeit data suggest levels below current national guidance [36]. Variability in organisational physiotherapy staffing models has also been reported in other settings including trauma centres [71] and primary care [72], with consequences including impact on individual and team-level clinician caseloads, profile of individual patient treatment plans, quality of patient care and clinician workload pressures – findings echoed in the current study. Our findings also suggest that fragmented patient care and therefore potentially poorer outcomes can arise because of uncoordinated MDT working. Siloed working, lack of collaboration and ineffective communication are also recognised as key barriers in the delivery of acute rehabilitation practice [73] indicating organisational factors are not unique to physiotherapy-delivered ACT.

### Methodological critique

We followed rigorous JBI methodology and reported in accordance with updated JBI PRISMA-ScR guidelines [20], ensuring a systematic and transparent approach for this review. The protocol was registered in advance enhancing transparency and reducing risk of reporting bias, and with no subsequent amendments. Search strategies were developed with expert input to maximise identification of potentially relevant search results. To maximise inclusivity, studies published in any language were included, albeit all final eligible studies were in English. Screening and data extraction processes were piloted and completed independently and in duplicate. Whilst data extraction focused on direct themes from primary studies, this was not possible for five studies. Nonetheless, relevant text-based data from these studies were extracted and integrated into the narrative synthesis and the refinement of theme descriptors, ensuring a comprehensive and inclusive representation of the available evidence. Formal quality appraisal of included studies was not undertaken, in keeping with established scoping review methodology [20].

This review focused on the hospital setting, but other relevant literature may exist in out-of-hospital contexts. Most included studies were conducted in ICU populations, with the remaining two studies involving mixed populations of ICU and ward patients. Including studies across different hospital settings (ICU and ward) may limit the specificity of findings, as ACT may be applied differently depending on the clinical and environmental context. However, this broad approach allowed for a more comprehensive synthesis of the current evidence and reflects the real-world transitions patients often experience during hospital admission.

Finally, although the review sought to include family members’ perspectives on ACT, no eligible studies were identified. This represents an important gap in the literature and highlights the need for future research that incorporates family or caregiver experiences, particularly given their potential role in supporting ACT practices. In addition, the overall evidence base was limited, with only 11 studies meeting the inclusion criteria. These studies represented five different study designs, which aligns with the remit of a scoping review to capture a broad and inclusive range of evidence [20]. However, despite this methodological inclusivity, only two included studies represented patient experiences, and one of these was an opinion piece rather than an empirically investigated study; consequently the narrative synthesis drew predominantly on healthcare professional perspectives. Together these factors further narrow the depth of insight available regarding patient experience in the hospital setting.

### Conclusion

This scoping review identified four themes relating to experiences of ACT delivery in the hospital setting. Current literature is dominated by physiotherapists’ perspectives and ICU contexts, with no studies capturing family experiences and limited insights from patients or the wider MDT in other hospital environments. To fully understand experiences of ACT there is a need for future research to adopt a broader, stakeholder-informed approach across all hospital settings. Such work is essential to meaningfully contribute to clinical guidelines, optimise care delivery and inform future trials that better reflect real-world practice.

## Supplementary material

**Please note:** supplementary material is not edited by the Editorial Office, and is uploaded as it has been supplied by the author. Supplementary material 00140-2026.SUPPLEMENT
