# Supplementary material for: Patient, family member and healthcare professionals’ experiences of physiotherapy airway clearance techniques during hospital admission: a scoping review
Source: ERJ Open Res. 2026 Sep 1;12(5):00140-2026. doi: 10.1183/23120541.00140-2026 (PMC13531308; doi:10.1183/23120541.00140-2026)
Supplement: Supplementary file 1 [file 00140-2026.SUPPLEMENT.pdf]

Patient, family member, and healthcare professional experiences of physiotherapy airway clearance techniques during hospital admission: a scoping review

SUPPLEMENTARY MATERIAL

1. PRISMA-ScR Checklist with associated changes by JBI p2-6

2. Search strategy p7-8

3. Extracted data p9

4. Excluded studies and reason for exclusion p10-13

5. Analysis – Interpretive descriptors and overarching themes p14-19

1. PRISMA-ScR Checklist with associated changes by JBI

The completed PRISMA-ScR Checklist, which outlines how each item was addressed in the scoping review.

SECTION	ITEM	PRISMA-ScR CHECKLIST ITEM	CHANGES TO CONSIDER SINCE PRISMA 2020	REPORTED ON PAGE #
	**TITLE**			
Title	1	Identify the report as a scoping review.		1
	**ABSTRACT**			
Structured summary	2	Provide a structured summary that includes (as applicable): background, objectives, eligibility criteria, sources of evidence, charting methods, results, and conclusions that relate to the review questions and objectives.	Use the abstract reporting checklist (see Item 2 in PRISMA 2020)	2
	**INTRODUCTION**			
Rationale	3	Describe the rationale for the review in the context of what is already known. Explain why the review questions/objectives lend themselves to a scoping review approach.		3
Objectives	4	Provide an explicit statement of the questions and objectives being addressed with reference to their key elements (e.g., population or participants, concepts, and context) or other relevant key elements used to conceptualize the review questions and/or objectives.		4
	**METHODS**			
Protocol and registration	5	Indicate whether a review protocol exists; state if and	Report any protocol amendments (see item 24 in PRISMA 2020)	3-4

SECTION	ITEM	PRISMA-ScR CHECKLIST ITEM	CHANGES TO CONSIDER SINCE PRISMA 2020	REPORTED ON PAGE #
		where it can be accessed (e.g., a Web address); and if available, provide registration information, including the registration number.		
Eligibility criteria	6	Specify characteristics of the sources of evidence used as eligibility criteria (e.g., years considered, language, and publication status), and provide a rationale.		4
Information sources*	7	Describe all information sources in the search (e.g., databases with dates of coverage and contact with authors to identify additional sources), as well as the date the most recent search was executed.		4
Search	8	Present the full electronic search strategy for at least 1 database, including any limits used, such that it could be repeated.	Include the full search strategies for **all** databases, registers, and websites (see item 7 in PRISMA 2020)	Supplementary material - 2
Selection of sources of evidence†	9	State the process for selecting sources of evidence (i.e., screening and eligibility) included in the scoping review.	Describe if automation tools were used for study selection (see item 8 in PRISMA 2020)	4<br>Figure 1
Data charting process‡	10	Describe the methods of charting data from the included sources of evidence (e.g., calibrated forms or forms that have been tested by the team before their use, and whether data charting was done independently	If outcomes were included, describe how they were defined and which results were sought (see item 10 in PRISMA 2020)	4-5

SECTION	ITEM	PRISMA-ScR CHECKLIST ITEM	CHANGES TO CONSIDER SINCE PRISMA 2020	REPORTED ON PAGE #
		or in duplicate) and any processes for obtaining and confirming data from investigators.		
Data items	11	List and define all variables for which data were sought and any assumptions and simplifications made.		Supplementary material - 3
Critical appraisal of individual sources of evidence§	12	If done, provide a rationale for conducting a critical appraisal of included sources of evidence; describe the methods used and how this information was used in any data synthesis (if appropriate).		N/A
Synthesis of results	13	Describe the methods of handling and summarizing the data that were charted.		4-5
	**RESULTS**			
Selection of sources of evidence	14	Give numbers of sources of evidence screened, assessed for eligibility, and included in the review, with reasons for exclusions at each stage, ideally using a flow diagram.	Use the updated PRISMA 2020 flow diagram, which has optional boxes for review updates, as well as studies that were identified through means other than searching databases /registers and cite any studies that appeared to meet the inclusion criteria but were excluded (see item 16 in PRISMA 2020)	5 Figure 1
Characteristics of sources of evidence	15	For each source of evidence, present characteristics for which data were charted and provide the citations.		5-6
Critical appraisal within sources of evidence	16	If done, present data on critical appraisal of included sources of evidence (see item 12).		N/A
Results of individual	17	For each included source of evidence,		17-23, Tables 2 and 3

SECTION	ITEM	PRISMA-ScR CHECKLIST ITEM	CHANGES TO CONSIDER SINCE PRISMA 2020	REPORTED ON PAGE #
sources of evidence		present the relevant data that were charted that relate to the review questions and objectives.		
Synthesis of results	18	Summarize and/or present the charting results as they relate to the review questions and objectives.		6-10<br>22-23, Table 3<br>Supplementary material - 5
	**DISCUSSION**			
Summary of evidence	19	Summarize the main results (including an overview of concepts, themes, and types of evidence available), link to the review questions and objectives, and consider the relevance to key groups.		9-14
Limitations	20	Discuss the limitations of the scoping review process.		13-14
Conclusions	21	Provide a general interpretation of the results with respect to the review questions and objectives, as well as potential implications and/or next steps.		14
	**FUNDING**			
Funding	22	Describe sources of funding for the included sources of evidence, as well as sources of funding for the scoping review. Describe the role of the funders of the scoping review.	Report conflicts of interest (see item 26 in PRISMA 2020)	23
**OTHER CONSIDERATIONS**<br>*In addition, a new item was included in PRISMA 2020, which recommends reporting where data and other materials from the review are publicly available (see item 27 in PRISMA 2020), which can be included when reporting a scoping review.[1]*				

SECTION	ITEM	PRISMA-ScR CHECKLIST ITEM	CHANGES TO CONSIDER SINCE PRISMA 2020	REPORTED ON PAGE #
Availability of data, code and other materials	27	Report which of the following are publicly available and where they can be found: template data collection forms; data extracted from included studies; data used for all analyses; analytic code; any other materials used in the review.		Template data collection form and accompanying data charting information sheet can be found online: https://doi.org/10.17605/OSF.IO/P4XJM<br><br>Supplementary material - 3 and 5

2. Search strategy

The strategies below show the search used in Ovid MEDLINE (All) and ProQuest Dissertations & Theses Global. These were adapted as appropriate for each database searched.

Search strategy for database: Ovid MEDLINE(R) ALL Search strategy

1. experience.mp.
2. views.mp.
3. Perception/
4. Clinical Reasoning/ or Clinical Decision-Making/
5. Attitude/
6. Behavior/
7. behaviour.mp.
8. Observation/
9. opinion.mp.
10. airway clearance techniques.mp.
11. airway clearance.mp.
12. Mucociliary Clearance/
13. lung clearance.mp.
14. Physical Therapists/ or physiotherapy.mp.
15. Respiratory Therapy/ or chest physiotherapy.mp. or Physical Therapy Modalities/
16. respiratory physiotherapy.mp.
17. manual assisted cough.mp.
18. Cough/
19. Drainage, Postural/
20. manual techniques.mp.
21. Percussion/
22. rib springs.mp.
23. Vibration/ or expiratory vibrations.mp.
24. Chest Wall Oscillation/
25. hyperinflation.mp.
26. manual hyperinflation.mp.
27. mhi.mp.
28. ventilator hyperinflation.mp.
29. vhi.mp.
30. Intermittent Positive-Pressure Breathing/

31. manual insufflation exsufflation.mp.

32. Patient Positioning/ or positioning.mp.

33. hospitalized.mp. or Hospitalization/

34. hospitalised.mp.

35. Hospitals/

36. ward.mp.

37. Intensive Care Units/ or icu.mp. or Critical Care/

38. hdu.mp.

39. high dependency unit.mp.

40. Inpatients/

41. acute care.mp.

42. After-Hours Care/ or on-call.mp.

43. 1 or 2 or 3 or 4 or 5 or 6 or 7 or 8 or 9

44. 33 or 34 or 35 or 36 or 37 or 38 or 39 or 40 or 41 or 42

45. 10 or 11 or 12 or 13 or 14 or 15 or 16 or 17 or 18 or 19 or 20 or 21 or 22 or 23 or 24 or 25 or 26 or 27 or 28 or 29 or 30 or 31 or 32

46. 43 and 44 and 45

Search strategy for grey literature: ProQuest Dissertations & Theses Global

Keywords: "airway clearance" AND (hospitalised or hospitalized) AND experience

3. Extracted data

This is the template used to extract and chart data from the included studies in accordance with the reviews objectives.

**Study characteristics**	• Author<br>• Year of publication<br>• Title	• Source<br>• Country of study<br>• Type of evidence source	• Study methodology<br>• Data collection method<br>• Study objective(s) / aim(s)
**Contextual information**	• Setting<br>• Population type<br>• Sample size	• Sex/age range of participants<br>• Diagnosis or health condition	
**Key details**	• Main outcome	• Themes related to airway clearance techniques	

4. Excluded studies and reason for exclusion

This records the studies reviewed and subsequently excluded at full text stage, along with the specific reason for each exclusion, in line with the eligibility criteria defined for the review.

No	Author, Year	Reason for exclusion
1.	Adler 1997	Wrong concept
2.	Al-Nassan 2023	Wrong study design
3.	Aldhuhoori 2021	Wrong study design
4.	Alqahtani 2020	Wrong study design
5.	Asante 2024	Wrong study design
6.	Baidya 2016	Wrong study design
7.	Battaglini 2020	Wrong study design
8.	Benson	Full text not available
9.	Bisht 2013	Wrong study design
10.	Boynton 2018	Full text not available
11.	Bradley 2023	Wrong concept
12.	Carruthers 2024	Wrong concept
13.	Carta 2019	Full text not available
14.	Cathcart 2016	Full text not available
15.	Corner 2019	Wrong concept
16.	Curtis 1993	Wrong study design
17.	deZwart 2023	Wrong concept
18.	Denehy 2018	Wrong concept
19.	Dijkstra 2024	Wrong concept
20.	Dugernier 2021	Wrong context
21.	Eriksen 2018	Full text not available
22.	Felten-Barentsz 2020	Wrong study design
23.	Gibson 2020	Full text not available
24.	Goggin 2011	Full text not available
25.	Gorman 2010	Wrong study design
26.	Gosselink 2008	Wrong context
27.	Gupte 2016	Wrong concept
28.	Hind 1999	Wrong concept

29.	Igwesi-Chidobe 2022	Wrong concept
30.	Jiandani 2021	Wrong concept
31.	Jiandani 2020	Wrong study design
32.	Johnson 2021	Wrong concept
33.	Jones 2001	Wrong study design
34.	Jones 1992	Wrong study design
35.	Kumar 2007	Wrong study design
36.	Lawler 2015	Wrong concept
37.	Le Neindre (#264)	Full text not available
38.	Le Neindre (#451)	Wrong study design
39.	Li 2021	Wrong study design
40.	Lim 2014	Full text not available
41.	Liput 2016	Wrong study design
42.	Liyanage 2024	Wrong study design
43.	Marti 2020	Wrong study design
44.	Matilde 2018	Wrong study design
45.	Mehta 2022	Full text not available
46.	Mihaka	Full text not available
47.	Mirza 2024	Wrong study design
48.	Moore 2015	Wrong study design
49.	Morrison 2022	Wrong study design
50.	Munakomi 2019	Wrong concept
51.	Naz 2021	Wrong context
52.	Ntinga 2020	Wrong concept
53.	O'Connor 2021	Wrong context
54.	O'Donnell 2019	Full text not available
55.	Oliveira 2016	Wrong population
56.	Opdekamp	Full text not available
57.	Osadnik 2013	Wrong study design
58.	Palacios-Cena 2021	Wrong concept
59.	Park 2023	Wrong study design
60.	Partridge 2007	Wrong study design
61.	Peralta	Full text not available

62.	Phan 2023	Wrong concept
63.	Plani 2017	Wrong concept
64.	Ramachandran 2020	Wrong concept
65.	Ramalho 2024	Wrong context
66.	Redgrave 2020	Full text not available
67.	Rees 2023	Wrong concept
68.	Rhodes-Eve	Full text not available
69.	Ricci 2024	Wrong concept
70.	Rose 2016 #118	Wrong study design
71.	Rose 2022 (#5)	Wrong concept
72.	Rose 2022 (#99)	Wrong concept
73.	Sabadosa 2012	Wrong study design
74.	Salvitti 2020	Wrong concept
75.	SanchezRomero 2022	Wrong context
76.	Smith 2007	Wrong concept
77.	Smith 2010	Wrong concept
78.	Sottile 2015	Wrong study design
79.	Steenbruggen 2022	Wrong concept
80.	Stewart 2018	Full text not available
81.	Swaiss 2004	Full text not available
82.	Swingwood 2023	Full text not available
83.	Tan 2017	Wrong concept
84.	Thomas 2008	Wrong concept
85.	Trojman 2023	Wrong study design
86.	Twose 2021	Wrong concept
87.	vanDelft 2021	Wrong study design
88.	vanderLee 2024	Wrong study design
89.	vanWilligen 2020	Wrong concept
90.	Viloria 2023	Wrong study design
91.	Wang 2020	Wrong concept
92.	Raios 2027	Wrong study design
93.	Moran 2005	Wrong study design
94.	Patterson 2005	Wrong study design

95.	Thackray 2017	Wrong context
96.	Franks 2022	Wrong population
97.	Siewers 2013	Wrong population
98.	Khalifa 2022	Wrong study design
99.	Saragosa 2024	Wrong concept
100.	Güngen 2017	Wrong study design
101.	Tomasz 2020	Full text not available
102.	AkhavanAttar 2025	Wrong concept
103.	Cheng 2022	Wrong study design
104.	Boyd 2025	Wrong concept
105.	Battaglini 2025	Wrong study design
106.	Wolfensberger 2023	Wrong study design
107.	Karachi 2025	Wrong concept
108.	Marques 2024	Wrong study design
109.	Huang 2025	Wrong concept
110.	Kromrey 2025	Wrong population
111.	Massey 2025	Wrong context
112.	Stabile 2024	Wrong population
113.	Fernández Rodríguez 2025	Wrong study design
114.	Çınar 2019	Wrong study design
115.	AshrafMegahed 2025	Wrong study design
116.	El-Kass 2024	Wrong study design
117.	Hodgson 2022	Wrong study design
118.	Barutçu 2019	Wrong study design
119.	Taniguchi 2022	Wrong study design
120.	Carruthers 2024	Wrong concept
121.	Baker 2025	Wrong study design

Study eligibility criteria using the Population-Concept-Context framework: Population - adults ≥16 years receiving healthcare professional-delivered ACT in hospital, family members observing/assisting ACT delivery, or healthcare professionals involved in ACT delivery; Concept - experiences or perspectives of delivering/receiving physiotherapy ACT (positioning, hyperinflation, manual techniques, mechanical insufflation-exsufflation, with saline instillation/suction as adjuncts); Context - hospital setting. Studies failing to address any of these criteria were excluded from the review.

5. Analysis – Interpretive descriptors and overarching themes

This table presents the analysis of studies included in the narrative synthesis. For each study, the author, year and country are listed alongside the population studied, themes related to ACT identified by the original authors, and interpretive descriptors and overarching themes agreed by the study team. For studies not reporting primary themes specific to ACT, relevant data were extracted, and overarching themes were assigned to ensure consistency and integration into the narrative synthesis.

Author, Year, Country	Population studied	Themes reported in study	Interpretive descriptor	Overarching theme
Cakmak, 2019 [2] Turkey	Healthcare professionals<br>• Physiotherapists working in the ICU who are part of the Turkish Physiotherapy Association network (n=65)	No primary themes specific to ACT.<br>Study reported themes related to barriers to ICU physiotherapy practice broadly.	Not applicable	Knowledge, resources, and skills<br>Organisational factors
Connolly, 2020 [3] UK	Healthcare professionals<br>• Physiotherapists with 3–27 years critical care experience; 10 worked in general (mixed medical, surgical, trauma, severe respiratory failure services) ICUs, 4 in cardiac ICUs, and 1 in a neurological ICU; ICU bed capacity ranged 16–54 beds (n=15)	Repertoire of airway clearance techniques	Adaption of technique to patient	Decision-making process
		Staffing and skillset	Impact of staffing across 7 days and skill levels	Organisational factors
		Commencing respiratory physiotherapy	Individualised assessment of patient	Decision-making process
		Technique selection	Sources of information to inform decision making	Decision-making process
		Determining effectiveness	Effectiveness and outcomes	Perceived effects and Outcomes

Eggmann, 2021 [4] Switzerland	Healthcare professionals<br>• Physical therapists treating patients with COVID-19 during their acute care hospital stay (n=not reported)	No primary themes, study was a case report series.	Not applicable	Decision-making process<br>Knowledge, resources, and skills
Karachi, 2023 [5] South Africa	Patients<br>• Admitted for emergency surgery and trauma, with a median age of 44 years and median ICU length of stay (LOS) of six days (n=18)	No primary themes specific to ACT.<br>Study reported themes related broadly to physiotherapy practice.	Not applicable	Perceived effects and Outcomes<br>Knowledge, resources, and skills
Raios, 2024 [6] Australia	Healthcare professionals<br>• Physiotherapists with a mean age of 33.5 years, 89% were female with a median 7.5 years of ICU experience at six metropolitan health services in Victoria, Australia (n=27)	(i) physiotherapists' involvement in care was highly variable	Impacting factors (organisational i.e. staffing, culture, exposure to patients)	Organisational factors
		(ii) physiotherapists were not aware of existing evidence or guidelines for the care of people who are potential donors and followed usual practices	Lack of physio knowledge of evidence base	Knowledge, resources, and skills
		(iii) a consistent vision of the physiotherapy role was lacking	Physio opinions on role of physiotherapy	Organisational factors
		(iv) physiotherapists' engagement with the team routinely involved in care of people who are	MDT views / membership	Organisational factors

		potential donors varied considerably		
		(v) physiotherapists faced practice challenges associated with delivering care to potential donors	Physiotherapist barriers and family barriers	Knowledge, resources, and skills
		(vi) several enablers could support a role for physiotherapy in this patient population.	Facilitators	Organisational factors
Stilma, 2023 [7] Netherlands	Healthcare professionals with experience of MI-E (n=35)<br>• ICU nurse (n=7)<br>• ICU nurse with additional 14-month ventilation course (n=9)<br>• Physicians (intensivists) (n=3)<br>• Physiotherapists (n=6)<br>• Clinical nurse specialist (n=2)	(1) knowledge	Limited evidence base	Knowledge, resources, and skills
		(2) beliefs	Safety of ACT	Decision-making process
		(3) clinical decision-making	MDT collaboration and their beliefs about ACT	Organisational factors
		(4) future adoption	MDT beliefs	Organisational factors
Van der Lee, 2017 [8] Australia	Healthcare professionals<br>• Physiotherapists across 88 Australian public and private hospitals, with a minimum of 5 years clinical experience and a minimum 12 months experience as a	Respiratory treatment techniques	Rationale for ACT choice	Decision-making process

	senior physiotherapist working in an adult ICU within the last 5-years (n=75)			
Van der Lee, 2018 [9] Australia	Healthcare professionals<br>• Physiotherapists with a minimum of 5 years as a qualified physiotherapist and a minimum of 3 years of experience in critical care. Participants represented clinical, academic and specialist roles. (n=29)	Domain 1—physiotherapy assessment	Physio assessment for timely ACT intervention	Decision-making process
		Domain 2—patient selection and prioritization	Skill and knowledge of physio	Knowledge, resources, and skills
		Domain 3—positioning and entral feeds	Patient safety associated with ACT	Decision-making process
		Domain 5—MCWT: manual chest wall techniques	Lack of evidence base and clinical rationale	Decision-making process
		Domain 6 - NSI	Lack of evidence base and clinical rationale	Decision-making process
		Domain 7 – active exercise and mobilisation	Rationale for ACT choice	Decision-making process
Van der Lee, 2020 [10] Australia	Healthcare professionals with more than 5 years' experience and working in an Australian level 2 or 3 ICU<br>• Physiotherapists (n=16)<br>• Nurses (n=4)<br>• Consultant intensivists (6)	1. Teamwork and communication	MDT communication	Organisational factors
		2. Patient safety	Patient instability and safety	Decision-making process
		3. Culture	Organisational difference in clinical practice	Organisational factors
Bellone, 2025 [11] Italy	Patients	No primary themes specific to ACT.<br>Study is an opinion paper.	Not applicable.	Decision-making process

Solares-Mogollón, 2025 [12]<br>Spain	Healthcare professionals from an ICU, with at least 2 years clinical experience and who currently work or have worked in coordination<br>with physiotherapists within the ICU.<br>• Physiotherapist (n=7)<br>• Nursing (n=7)<br>• Internal Medicine Specialist (n=7)	No primary themes specific to ACT.<br>Study reported themes related to the strengths and barriers of administering a physiotherapy treatment to patients admitted to an ICU.	Not applicable.	Perceived effects and Outcomes<br>Organisational factors

REFERENCES

1. Page MJ, McKenzie JE, Bossuyt PM, Boutron I, Hoffmann TC, Mulrow CD, Shamseer L, Tetzlaff JM, Akl EA, Brennan SE, Chou R, Glanville J, Grimshaw JM, Hrobjartsson A, Lalu MM, Li T, Loder EW, Mayo-Wilson E, McDonald S, McGuinness LA, Stewart LA, Thomas J, Tricco AC, Welch VA, Whiting P, Moher D. The PRISMA 2020 statement: an updated guideline for reporting systematic reviews. *BMJ* 2021: 372: n71.
2. Cakmak A, Ince DI, Saglam M, Savci S, Yagli NV, Kutukcu EC, Ozel CB, Ulu HS, Arikan H. Physiotherapy and Rehabilitation Implementation in Intensive Care Units: A Survey Study. *Turkish Thoracic Journal* 2019: 20(2): 114-119.
3. Connolly B, Barclay M, Blackwood B, Bradley J, Anand R, Borthwick M, Chikhani M, Dark P, Shyamsundar M, Warburton J, McAuley D, O'Neill B. Airway clearance techniques and use of mucoactive agents for adult critically ill patients with acute respiratory failure: A qualitative study exploring UK physiotherapy practice. *Physiotherapy* 2020: 108: 78-87.
4. Eggmann S, Kindler A, Perren A, Ott N, Johannes F, Vollenweider R, Balma T, Bennett C, Silva IN, Jakob SM. Early Physical Therapist Interventions for Patients With COVID-19 in the Acute Care Hospital: A Case Report Series. *Physical Therapy* 2021: 101(1): 04.
5. Karachi F, van Nes MB, Gosselink R, Hanekom S. Patient perceptions of ICU physiotherapy: 'Your body needs to go somewhere to be recharged ... '. *Southern African Journal of Critical Care* 2023: 39(3): e1092.

6. Raios C, Keating JL, Skinner EH, Opdam HI, Haines KJ. Exploring current physiotherapy practices in the care of people who are potential lung donors: A qualitative study. *Australian Critical Care* 2024: 37(4): 539-547.
7. Stilma W, Verweij L, Spek B, Scholte Op Reimer WJM, Schultz MJ, Paulus F, Rose L. Mechanical insufflation-exsufflation for invasively ventilated critically ill patients-A focus group study. *Nursing in Critical Care* 2023: 28(6): 923-930.
8. van der Lee L, Hill AM, Patman S. A survey of clinicians regarding respiratory physiotherapy intervention for intubated and mechanically ventilated patients with community-acquired pneumonia. What is current practice in Australian ICUs? *J Eval Clin Pract* 2017: 23(4): 812-820.
9. van der Lee L, Hill AM, Patman S. Expert consensus for respiratory physiotherapy management of mechanically ventilated adults with community-acquired pneumonia: A Delphi study. *J Eval Clin Pract* 2018: 25(2): 230-243.
10. van der Lee L, Hill AM, Patman S. Clinical validation of expert consensus statements for respiratory physiotherapy management of invasively ventilated adults with community-acquired pneumonia: A qualitative study. *Intensive Crit Care Nurs* 2020: 60: 102854.
11. Bellone F, Tisano A, Leonardi G, Amato D, Borzelli D, Santoro G, Alito A, Cucinotta F, Portaro S. Lesson from COVID-19-Adapting Respiratory Rehabilitation Through Early Multidisciplinary Care: An Opinion Paper from Retrospective Data. *Journal of Clinical Medicine* 2025: 14(11) (no pagination).
12. Solares-Mogollon A, Cuesta-Barriuso R. Strengths and Weaknesses of Physiotherapy in the Daily Work of an Intensive Care Unit: A Qualitative Study. *Journal of Clinical Medicine* 2025: 14(7) (no pagination).
